# Supplementary material for: A Small Step, a Giant Leap: Somatic Hypermutation of a Single Amino Acid Leads to Anti-La Autoreactivity
Source: Int J Mol Sci. 2021 Nov 7;22(21):12046. doi: 10.3390/ijms222112046 (PMC8584381; doi:10.3390/ijms222112046)
Supplement: Supplementary file 1 [file ijms-22-12046-s001.zip › ijms-1413538-supplementary.pdf]

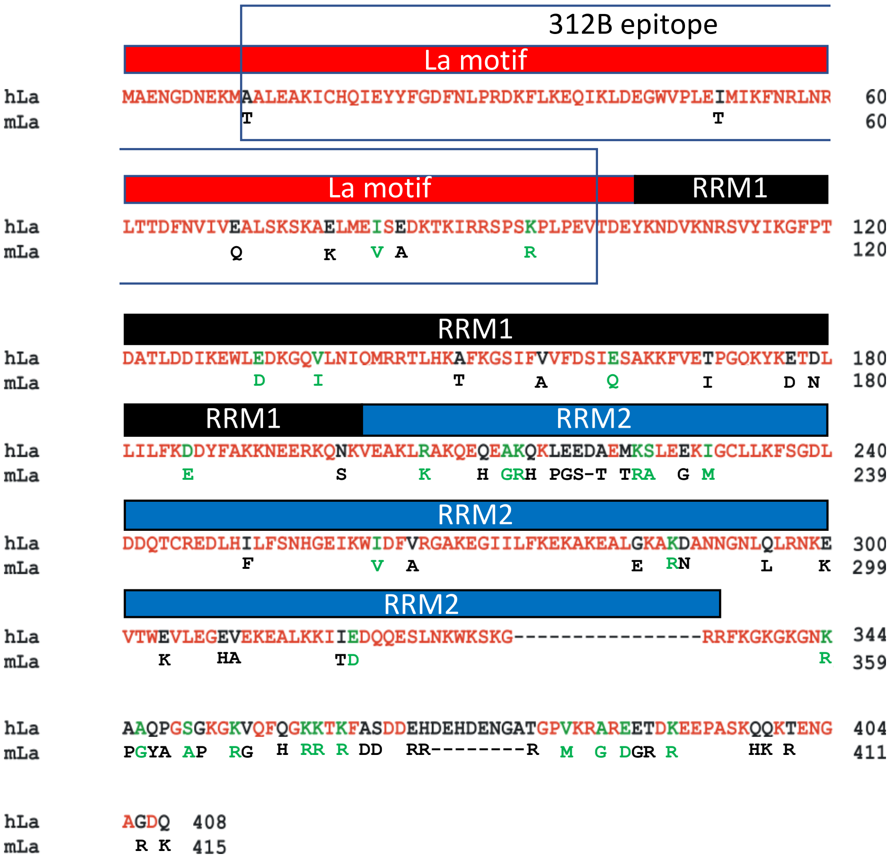

Supplemental Figure S1. Comparison of human and mouse La protein. La protein is highly conserved during evolution which includes human (hLa) and mouse La protein (mLa). The sequence homology is highest in the La motif and the RRM1 domain. The RRM2 contains two species specific sequence regions [52]. The discontinuous epitope recognized by the anti-La mab 312B part of the La motif (blue box).
